# Deterioration in Renal Function in Patients With a Fontan Circulation and Association With Mortality

**DOI:** 10.1016/j.jacadv.2024.101399

**Published:** 2024-11-19

**Authors:** Gaston van Hassel, Dion Groothof, Johannes M. Douwes, Elke S. Hoendermis, Eryn T. Liem, Tineke P. Willems, Tjark Ebels, Adriaan A. Voors, Stephan J.L. Bakker, Rolf M.F. Berger, Joost P. van Melle

**Affiliations:** a Center for Congenital Heart Diseases, Paediatric Cardiology, Beatrix Children’s Hospital, University of Groningen, University Medical Center Groningen, Groningen, the Netherlands; b Division of Nephrology, Department of Internal Medicine, University Medical Center Groningen, University of Groningen, Groningen, the Netherlands; c Department of Cardiology, University Medical Center Groningen, University of Groningen, Groningen, the Netherlands; d Department of Radiology, University Medical Center Groningen, University of Groningen, Groningen, the Netherlands; e Department of Cardiothoracic Surgery, University Medical Center Groningen, University of Groningen, Groningen, the Netherlands

**Keywords:** congenital, Fontan, longitudinal, renal function, univentricular heart

## Abstract

**Background:**

Renal dysfunction is a well-established risk factor in cardiovascular disease, but little is known about the prevalence and factors associated with deterioration in renal function in patients with a Fontan circulation.

**Objectives:**

The purpose of the study was to investigate the course and factors associated with deterioration in renal function in patients with a Fontan circulation and its association with mortality.

**Methods:**

This is a longitudinal study of patients with a Fontan circulation (n = 82), in which creatinine-based estimated glomerular filtration rate (eGFR_cr_) was measured over an 11-year time period. Cystatin C and N-terminal prohormone of brain natriuretic peptide (NT-proBNP) levels were measured at baseline. Renal dysfunction was defined as an eGFR <90 ml/min/1.73 m^2^. Factors associated with annual change in eGFR_cr_ were investigated with linear mixed-effect models and compared with data from a healthy Dutch cohort. The primary endpoint for the survival analyses was all-cause mortality. Associations between repeated eGFR_cr_ levels and the primary endpoint were assessed using a joint model.

**Results:**

The median age at baseline was 20 years (IQR: 14-27 years). Twelve percent of the cohort had renal dysfunction based on eGFR_cr_ and 24% based on cystatin C-based eGFR_cys_. During follow-up, eGFR_cr_ deteriorated on average by 1.36 ml/min/1.73 m^2^/year, which is faster than the healthy cohort. Higher baseline NT-proBNP z-scores were associated with a more rapid decline in eGFR_cr_. A larger decline in eGFR_cr_ was associated with all-cause mortality.

**Conclusions:**

Declines in eGFR_cr_ in patients with Fontan circulation are more rapid than in healthy individuals. Higher baseline NT-proBNP z-scores are associated with a more rapid deterioration of eGFR_cr_, and eGFR_cr_ deterioration is associated with mortality.

In the current era, patients born with a functionally univentricular heart are palliated with a Fontan circulation.^1^ In this circulation, the systemic venous return is redirected to the lungs, bypassing the subpulmonary ventricle. The absence of a subpulmonary ventricular pump to overcome pulmonary vascular resistance leads to a chronically elevated central venous pressure (CVP) and decreased cardiac output (CO), starting right after the Fontan operation.^2^ These altered hemodynamics of a Fontan circulation are generally well tolerated in early childhood. However, as a result of the chronic combination of nonphysiologically increased CVP, a nonpulsatile pulmonary blood flow, and a preload-deprived systemic ventricle, the Fontan circulation deteriorates over time. This deterioration, referred to as Fontan failure, is characterized by a gradually increasing CVP, decreasing CO, and a broad spectrum of complications including arrhythmias, thromboembolic disease, plastic bronchitis, protein-losing enteropathy, ascites, and Fontan-associated liver disease.^2^^,^^3^

Recently, renal dysfunction in Fontan patients has gained interest due to its significant association with quality of life and long-term adverse outcomes.^4^ The reported prevalence of renal dysfunction in the Fontan population varies between 10% and 50% depending on the study population and whether estimated glomerular filtration rate (eGFR) is calculated with creatinine or muscle mass-independent cystatin C.^5^ Previous studies on Fontan-associated nephropathy were limited to a cross-sectional setting, prohibiting an accurate evaluation of the course of renal function over time. Therefore, we aimed to quantify the changes in renal function in Fontan patients over time and assess factors associated with kidney function deterioration.

## Methods

### Study population, design, and data collection

In this study, we included 82 Fontan patients ≥10 years of age followed at the University Medical Center Groningen, the Netherlands. The original cohort was established in 2012 and has been described in detail previously.^6^^,^^7^ At the University Medical Center Groningen, Fontan patients undergo a regular and standardized follow-up, including cardiopulmonary exercise testing, cardiac magnetic resonance (CMR) examination, echocardiography, and venepuncture for laboratory measurements every 1 to 2 years. All included patients had a venepuncture for assessment of plasma creatinine levels at baseline and during follow-up per protocol. Baseline assessment of laboratory measurements, echocardiography, cardiopulmonary exercise testing, and CMR was performed in the period between 2012 and 2014. Demographic, clinical, and cardiac-related characteristics were extracted from patient records.

Written consent was acquired from all patients and/or their parents. The institutional ethics committee approved the conduct of this investigation (METc 2012080). This study was conducted in accordance with the principles outlined in the Declaration of Helsinki.

### Clinical outcomes

For this study, follow-up data were collected up to January 1, 2024. Data were collected from medical records. The endpoint was defined as all-cause mortality.

### Renal function

eGFR was calculated using the Chronic Kidney Disease Epidemiology Collaboration equation (CKD-EPI) for individuals aged 18 or older at baseline (hereafter referred to as “adults”) and the bedside Schwartz and CKiD equation for individuals younger than 18 years old (hereafter referred to as “children”).^8^^,^^9^ Renal dysfunction was defined as an eGFR <90 ml/min/1.73 m^2^ and was calculated using both serum creatinine (eGFR_cr_) and cystatin C (eGFR_cys_).^9^ For these analyses, plasma creatinine levels were measured using standard laboratory techniques (Roche Diagnostics). Follow-up creatinine levels were collected at multiple time points during this study, up to 11 years after baseline measurements. Serum cystatin C, a renal biomarker that is unaffected by nonrenal factors such as muscle mass, was measured from frozen serum (Gentian AS) that was collected at baseline.^10^ Additionally, plasma concentration of N-terminal prohormone of brain natriuretic peptide (NT-pro-BNP) was also measured at baseline.

### Statistical analyses

Baseline data are presented as mean ± SD, median (Q1-Q3), or number (percentage) depending on distribution. Normality was evaluated by visual inspection of histograms and Q-Q plots. All analyses were performed, and all figures were designed using the R statistical software (version 4.3.2). A 2-sided *P* < 0.05 was considered to indicate statistical significance.

In order to account for dependency of NT-proBNP on age, z-scores of NT-proBNP were used as previously described.^11^

We investigated the baseline prevalence of renal dysfunction in our cohort using both eGFR_cr_ and eGFR_cys_. In order to further investigate the prevalence and degree of renal dysfunction, both eGFR_cr_ and eGFR_cys_ were stratified according to CKD stages as defined by the Kidney Disease Improving Outcomes group.^12^

The overall annual changes in eGFR_cr_ were obtained using subject-specific slope estimates of a linear mixed model for eGFR_cr_ with random intercepts, random slopes for age, and their covariance. Fixed effects were specified for age, sex, serum cystatin C, and NT-proBNP z-scores. An appropriate random-effects structure was selected using likelihood ratio tests, which were based on mixtures of chi-square distributions.

Effects of covariates on changes in eGFR_cr_ were quantified using a linear mixed-effects model specifying random intercepts and random slopes of age per participant, as well as their covariance. Fixed effects were specified for serum cystatin C, sex, NT-proBNP z-scores, and their interaction with age. To allow for nonlinear evolutions of eGFR_cr_ over time, natural cubic splines with two degrees of freedom were used to model age (in both the fixed and random effects). Boundary knots were set to the 5th and 95th percentiles of age. Appropriate fixed- and random-effects structures were selected using likelihood ratio tests, based on mixtures of chi-square distributions where applicable. *P* values were corrected for multiple testing using the Bonferroni method. Model assumptions were validated by plotting marginal and conditional residuals against the fitted values and every covariate. Normality of the residuals was evaluated by inspection of Q-Q plots. Given the differences in recommended eGFR calculations between children and adults and the known limitations of the Schwartz equation in adolescents, we confined our analyses to the course of eGFR_cr_ from the age of 18 onward.

A joint model was fitted to assess the association between estimated eGFR_cr_ levels during follow-up, calculated using the repeated time-dependent eGFR_cr_ levels, and the specified endpoints. A joint model combines a linear mixed-effects model with a Cox proportional hazards model for the risk of the specified study endpoints. The linear mixed-effects model was fitted as previously described, but with follow-up time rather than age as the time variable. We combined the slope of the eGFR_cr_ trajectories in a joint model to assess its prognostic value and adjusted for age and sex. The results are presented as HR per 1-U decrease of eGFR_cr_ at any point in time with 95% CIs.

### Control population (longitudinal eGFR_cr_ measurements)

We compared the annual change in renal function in this cohort with reference values for eGFR from a previously published cross-sectional study consisting of a healthy Dutch population. This study included 6,097 (54% female) participants with a median age of 58 years ranging from 18 to 98 years. All patients in this study underwent one eGFR_cr_ measurement in order to provide reference values for eGFR_cr_.^13^

## Results

### Characteristics

Patient characteristics at baseline are presented in Table 1. The median age of the Fontan patients was 20 years (Q1-Q3: 14-27 years), and 51% were female. Sixty percent of the population was 18 years or older at baseline. The most frequent underlying cardiac diagnosis was tricuspid atresia (43%), followed by double inlet left ventricle (23%). The majority of our population had a lateral tunnel or an extracardiac conduit (54% and 28%, respectively), and 82% had a systemic left ventricular morphology. At baseline, 44%, 45%, and 11% of patients were classified as NYHA functional class I, II, and III, respectively. At baseline, 9 patients used medication that possibly could impact renal function. Five patients (6%) used angiotensin-converting enzyme inhibitors, and six patients (7%) used loop diuretics. Mean percentage of predicted peak oxygen uptake during exercise was 58% ± 14%. Median NT-proBNP was 112 (Q1-Q3: 62-268) ng/L. The median NT-proBNP z-score was 1.82 (Q1-Q3: 0.81-3.36). A z-score of 0 corresponded to NT-proBNP levels of 34 ng/L, and each increase of 1 SD (z-score = 1) equated to a 56.45 ng/L increase in NT-proBNP levels.

**Table 1:** Patient Characteristics at Baseline (N = 82)

Age, y	20 (14-27)
Female	42 (51)
LV morphology	67 (82)
Cardiac diagnosis	
Tricuspid atresia	35 (43)
Hypoplastic left heart syndrome	2 (2)
Double inlet left ventricle	19 (23)
Pulmunary atresia without VSD	10 (12)
AV septal defect/unbalanced VSD	11 (13)
Heterogenous anomalies	5 (6)
Fontan type	
Atrial pulmonary connection/Björk	15 (18)
Lateral tunnel	44 (54)
Extracardiac conduit	23 (28)
Pacemaker	14 (17)
NYHA functional class	
I	36 (44)
II	37 (45)
III	9 (11)
CPET (n = 73)	
pVO_2_ predicted, %	58.1 ± 13.6
Medication	
ACEI	5 (6)
ARB	0 (0)
Loop diuretics	6 (7)
NSAID	0 (0)
Echocardiography	
Ventricular function	
Good	33 (40)
Mildly impaired	39 (48)
Moderately impaired	10 (12)
Laboratory parameters	
Creatinine, μmol/L	63.0 (55.8-77.0)
Creatinine, mg/dL	0.71 (0.63-0.87)
eGFR_cr,_ mL/min/l.73 m^2^	108 ± 18
Cystatin C, mg/L	0.79 (0.69-0.93)
eGFR_cys,_ mL/min/l.73 m^2^	102 ± 22
Sodium, mmol/L	141.4 ± 2.0
Potassium, mmol/L	4.1 ± 0.3
Blood urea nitrogen, mmol/L	4.9 ± 1.7
ɣGT, U/L	59 (39-99)
NT-proBNP, ng/L	112 (62-268)
NT-proBNP, z-scores	1.82 (0.81-3.36)

ɣGT = ɣ-glutamyltransferase; ACEI = angiotensin-converting enzyme inhibitor; ARB = angiotensin II receptor blocker; AV = atrioventricular; CPET = cardiopulmonary exercise test; eGFR = estimated glomerular filtration rate; LV = left ventricle; NSAID = nonsteroidal anti-inflammatory drug; NT-proBNP = N-terminal prohormone of brain natriuretic peptide; NYHA = New York Heart Association; pVO_2_ = peak oxygen uptake; VSD = ventricular septal defect.

### Prevalence of renal dysfunction

**Figure 1:**
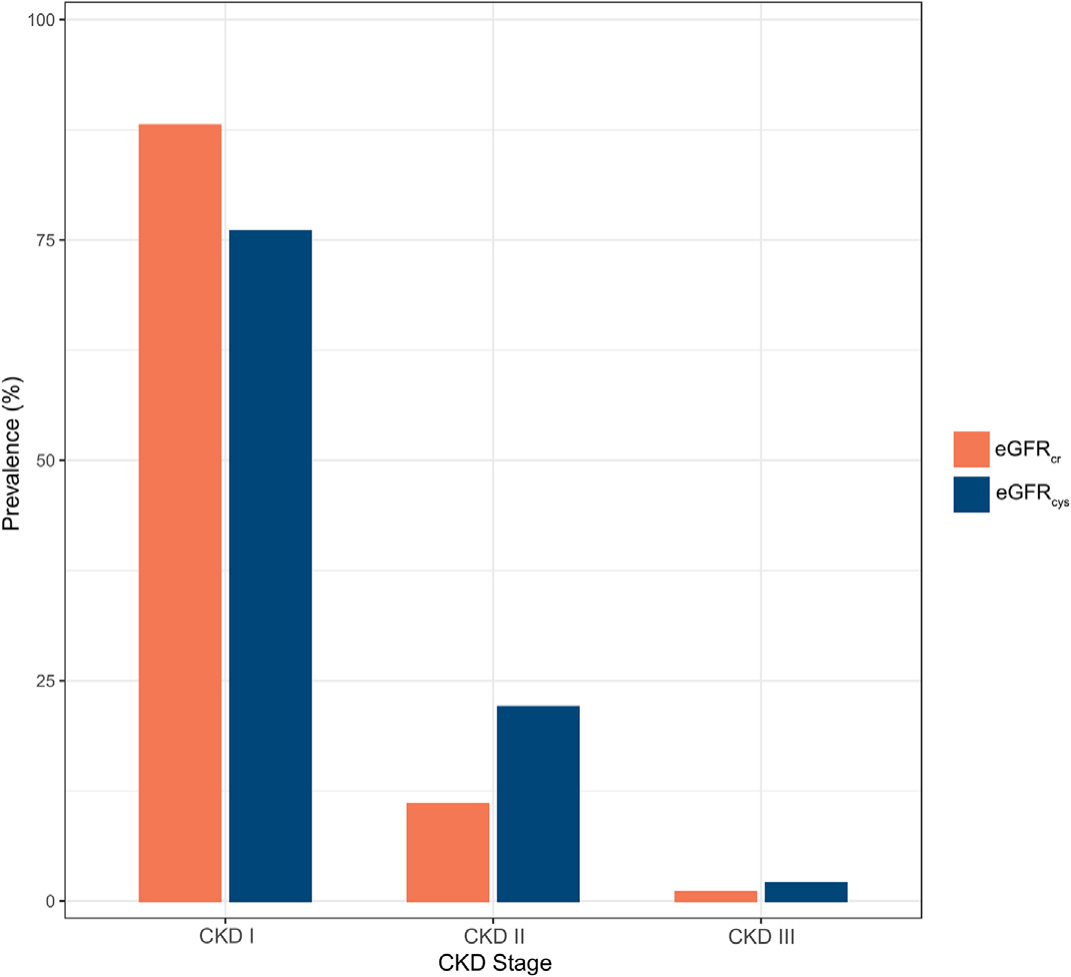
**Prevalence of CKD Stages at Baseline for the Entire Cohort** Creatinine-based eGFR (eGFR_cr_) is displayed as red and cystatin C-based eGFR (eGFR_cys_) is displayed as blue. CKD stage IV is not shown as no study participants were present in this specific group. CKD = chronic kidney disease; eGFR = estimated glomerular filtration rate.

At baseline, median plasma creatinine was 63.0 (Q1-Q3: 5.8-77.0) μmol/L (0.71 (Q1-Q3: 0.63-0.87) mg/dL) and median cystatin C concentration was 0.79 (0.69-0.93) mg/L. Baseline eGFR_cr_ was higher than eGFR_cys_ (108 ± 18 vs 102 ± 22 mL/min/1.73 m^2^, *P* < 0.001). When using eGFR_cr_, 10 patients (12%) had renal dysfunction. Using cystatin C for eGFR_cys_, 20 patients (24%) could be classified with renal dysfunction. There was no difference in use of nephrotoxic medication between patients with and without renal dysfunction (*P* = 0.07). When subdividing eGFR according to CKD stages, the prevalence of CKD stage I (eGFR >90 mL/min/1.73 m^2^) was 88% (n = 72) using eGFR_cr_ and 76% (n = 62) using eGFR_cys_. The prevalence of CKD stage II (eGFR 60-90 mL/min/1.73 m^2^) was 11% (n = 9) using eGFR_cr_ and 22% (n = 18) using eGFR_cys_. The prevalence of CKD stage III (eGFR 30-59 mL/min/1.73 m^2^) was 1% (n = 1) using eGFR_cr_ and 2% (n = 2) using eGFR_cys_ (Figure 1).

### Evaluation of the trajectory of renal function and associated factors

In our adult Fontan cohort, we found a mean annual change in eGFR_cr_ of −1.36 (95% CI: −1.65 to −1.06) mL/min/1.73 m^2^, which was greater than the annual change in eGFR_cr_ found in a healthy reference population (−0.86 mL/min/1.73 m^2^), despite the fact that eGFR_cr_ levels were approximately equal at the start of adulthood (Central Illustration).

**Figure 2:**
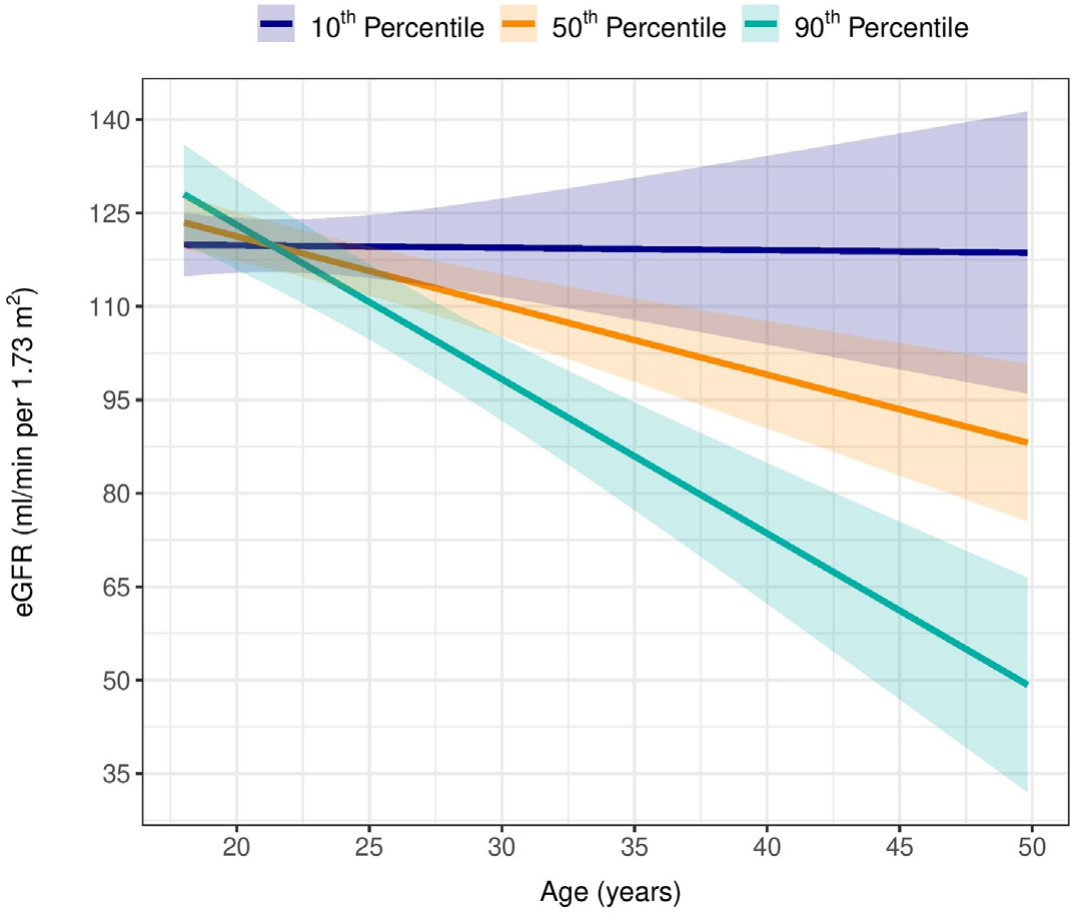
**Effect of Baseline NT-proBNP Z-Scores on Trajectories of eGFR**_**cr**_ The lines represent the conditional mean for the 10th, 50th, and 90th percentiles of NT-proBNP Z-scores for an 18-year-old male participant with all other covariates at their median value, obtained with linear mixed-effect model for eGFR_cr_. The shaded areas about the lines are the corresponding 95% pointwise CIs. eGFR_cr_ = estimated creatinine-based glomerular filtration rate; NT-proBNP = N-terminal prohormone of brain natriuretic peptide.

Table 2 shows effect estimates of the associations between repeatedly measured eGFR_cr_ and covariates. At each point during follow-up, mean eGFR_cr_ was 7.74 (95% CI: 2.51-12.97) mL/min/1.73 m^2^ higher in males than females. Further, for every mg/l increase in baseline serum cystatin C, the trajectory of eGFR_cr_ decreased by 15.96 (95% CI: 2.01-29.91), independent of other effects. Importantly, for each SD increase in baseline NT-proBNP z-score (ie, 56.45 ng/L), eGFR_cr_ decreased by an additional 0.51 (95% CI: −0.75 to −0.26) mL/min/1.73 m^2^/year (Figure 2).

**Table 2:** Effect Estimates From a Linear Mixed-Effects Model for the Outcome of eGFR_cr_ in Patients With a Fontan Circulation

	Β (95% CI)	*P* Value
Intercept	122.03 (99.00-145.07)	<0.001
Age, y	0.12 (−0.74 to 0.97)	0.9
Sex		
Female	Ref.	Ref.
Male	7.74 (2.51-12.97)	0.004
Cystatin C	−15.96 (−29.91 to −2.01)	0.03
NT-proBNP z-scores	10.80 (4.73-16.86)	0.01
Age ∗ NT-proBNP z-scores	−0.51 (−0.75 to −0.26)	0.005

The parameter NT-proBNP z-score refers to the main effect of NT-proBNP z-scores on the levels of eGFR_cr_. The interaction factor Age ∗ NT-proBNP z-scores refers to the main effect of NT-proBNP per year (ie, the slope of eGFR_cr_).

eGFR_cr_ = creatinine-based estimated glomerular filtration rate; NT-proBNP = N-terminal prohormone of brain natriuretic peptide.

**Central Illustration:**
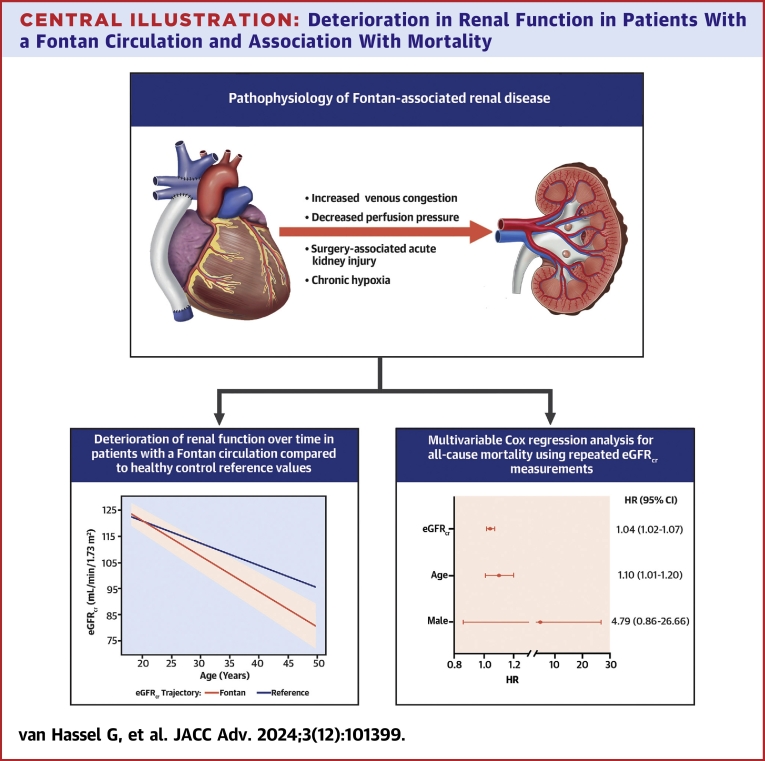
**Deterioration in Renal Function in Patients With a Fontan Circulation and Association With Mortality** Cardiorenal interactions in the Fontan circulation associated with renal dysfunction are depicted in the upper panel. The average deterioration of eGFR_cr_ over time in adult Fontan patients is compared to that of healthy individuals and depicted on the left. In this illustration, the orange line represents the predicted course of eGFR_cr_ during adulthood in our Fontan population, as obtained from the linear mixed-effect model. The shaded area around the line is the corresponding 95% CI. The black line depicts the mean change in eGFR_cr_ for a healthy 18-year-old individual. A forest plot for the multivariable Cox regression analysis of the longitudinal changes in eGFR_cr_ with all-cause mortality is depicted in the right panel. The HR for eGFR_cr_ corresponds to the HR per 1.0 mL/min/1.73 m^2^ decrease at any point in time. Created with BioRender.com. eGFR_cr_ = estimated creatinine-based glomerular filtration rate.

### Association between change in eGFR_cr_ and outcome

During the 11-year follow-up, eight patients died. The median age of the patients at the time of event was 33 years (Q1-Q3: 30-49 years). After adjustment for age and sex, the HR for all-cause mortality corresponding to a 1.0 mL/min/1.73 m^2^ decrease in eGFR_cr_ during follow-up was 1.04 (95% CI: 1.02-1.07; *P* = 0.01) (Central Illustration, Table 3).

**Table 3:** HR for All-Cause Mortality per 1 mL/min/1.73 m^2^ Decrease of eGFR_cr_ at Any Point in Time Using Repeated eGFR_cr_ Measurements in a Joint Model

	HR (95% CI)	*P* Value
eGFR_cr_	1.04 (1.02-1.07)	0.01
Age, y	1.10 (1.01-1.20)	0.03
Sex		
Female	Ref.	Ref.
Male	4.79 (0.86-26.66)	0.07

eGFR_cr_ = creatinine-based estimated glomerular filtration rate.

## Discussion:

To our knowledge, this is the first study to report the course of renal function over time in a wide age range of patients with a Fontan circulation. In adults aged 18 to 50 years, baseline measurements indicated a higher prevalence of renal dysfunction when assessed using cystatin C compared to creatinine. eGFR_cr_ was found to deteriorate faster in Fontan patients compared with healthy individuals, and higher baseline NT-proBNP z-scores were independently associated with a faster deterioration of eGFR_cr_. Lastly, deterioration of eGFR_cr_ was associated with mortality. These findings suggest that renal dysfunction is early and progressive in the Fontan population, and ongoing surveillance by clinicians is important.

### Prevalence of renal dysfunction

Few studies have investigated the prevalence of renal dysfunction in Fontan patients. Among those that have, prevalence numbers vary significantly, which can be attributed to several reasons.

The prevalence of renal dysfunction depends on the method of glomerular filtration rate (GFR) estimation and therewith the age of the participants. For example, Broda et al found a prevalence of 22% of renal dysfunction in a cohort consisting mostly of children with a Fontan circulation.^14^ In this study, eGFR_cr_ was determined by bedsides Schwartz equation, in which eGFR_cr_ is calculated by dividing the patients’ height by the concentration of circulating creatinine. However, as children age, muscle mass increases faster than height, which can lead to underestimation of eGFR_cr_, especially during adolescence.^15^, ^16^, ^17^ The fact that Broda et al found similar prevalences of renal dysfunction in children when compared to studies in adults might be explained by underestimation of eGFR_cr_ as a result of the use of the Schwartz equation in children.^18^, ^19^, ^20^ Notably, we found that, at the start of adulthood, eGFR_cr_ in patients with a Fontan circulation is similar to that of healthy individuals, which implies that renal dysfunction, as quantified with eGFR_cr_, is predominantly a long-term complication of the Fontan circulation that starts during early adulthood and gradually worsens over time.

In addition to age and the used GFR-estimating equation, eGFR is also determined by the biomarker used. In the current study, we found a 10% prevalence of renal dysfunction using eGFR_cr_ and 17% using eGFR_cys_. Serum creatinine, stemming from the spontaneous, nonenzymatic conversion of creatine and phosphocreatine, is the traditional marker for GFR estimation.^17^ However, its levels are dependent on multiple factors including age, sex muscle mass, and renal clearance. In contrast, cystatin C is marker of GFR and is unaffected by the aforementioned nonrenal factors.^21^ Accordingly, cystatin C is considered a more accurate biomarker to estimate GFR, especially in Fontan patients who tend to be shorter and have less muscle mass than would be expected for their size, which may result in an overestimation of eGFR_cr_.^22^ An accurate assessment of renal function is important for clinical decision-making, as an overestimation of eGFR could delay the diagnosis of CKD and hamper the identification of patients at risk for unfavorable outcomes.

### Evaluation of the trajectory of renal function and associated factors

An important finding of this study is the faster deterioration in renal function in adult patients with a Fontan circulation compared with a healthy reference population (Central Illustration). At the start of adulthood, eGFR_cr_ in adult Fontan patients is comparable to that of healthy individuals. However, as both populations age, eGFR_cr_ deteriorates faster in adults with a Fontan circulation.

Patients with a Fontan circulation are inherently exposed to various factors that could explain an accelerated deterioration of renal function, some of those already manifesting themselves prior to the completion of the Fontan circulation. Such patients often undergo a series of surgical interventions throughout their lives. These surgeries, while crucial for improving cardiovascular outcomes and prolonging life, present an inherent risk. Elevated preoperative pulmonary arterial pressures have been shown to be an independent risk factor for early postoperative acute kidney injury (AKI).^23^ Additionally, with each successive cardiac surgery a patient undergoes the risk of AKI further increases.^23^ Subsequently, each episode of AKI is associated with an increased risk of developing CKD later in life.^24^ This is particularly concerning given that multiple surgeries are required for the completion of a Fontan circulation. Therefore, patients with a Fontan circulation start their adulthood with an increased risk of developing CKD.

Next to these early challenges, several factors inherent to the Fontan circulation can further contribute to the accelerated renal function deterioration. Worsening hypoxia is a common feature of the Fontan physiology.^25^ Hypoxia in turn induces a state of chronic inflammation, which is often seen in patients with a Fontan circulation.^26^^,^^27^ This state of chronic inflammation subsequently forms a pathophysiological pathway that induces or results in the progression of CKD.^26^ In addition to worsening hypoxia, renal dysfunction may also be caused by a disproportionate reduction in renal perfusion. Renal perfusion pressure—the difference between mean systemic arterial pressure and CVP—is chronically decreased as a result of the elevated CVP that is inherent to the Fontan circulation. Reduced renal perfusion activates several compensatory mechanisms: the renin-angiotensin-aldosterone system, the sympathetic nervous system, and arginine vasopressin secretion, all of which result in fluid retention and subsequent venous congestion.^28^ This is particularly problematic in patients with a Fontan circulation who already experience venous congestion due to a chronically elevated CVP. In turn, this venous congestion further impairs renal function by reducing renal perfusion pressure and inducing direct damage to renal structures.^29^ Additionally, the persistent absence of a pulsatile pulmonary blood flow will, over time, increase pulmonary vascular resistance, thereby further decreasing CO and increasing CVP.^30^ This leads to a vicious cycle in which the kidneys are effectively wedged within these harmful hemodynamic disturbances, causing a deterioration of renal function over time.

Renal dysfunction is a well-established risk marker of clinical outcomes in cardiovascular disease. Previously, Opotowsky et al reported that a single baseline eGFR_cys_ measurement was associated with the composite outcome of all-cause mortality and nonelective cardiac hospitalization in patients with Fontan circulation. However, this study did not find a similar association when using eGFR_cr_.^31^ In the current study, using serial measurements of eGFR_cr_, we found that deterioration of eGFR_cr_ is associated with all-cause mortality. While such relationship has been described in patients with congestive heart failure, our study is the first to investigate this relationship in patients with Fontan circulation.^32^ As seen in the Central Illustration, renal dysfunction is an early and progressive manifestation of multiorgan failure that is caused by the abnormal physiology of the Fontan circulation. Consequently, due to its association with outcome, deterioration of renal function can potentially serve as an easily measurable indicator of end-organ damage and hence circulatory deterioration. Therefore, early detection of (progressive) renal dysfunction may play a vital role in ensuring a preventive strategy to improve long-term outcomes in patients with Fontan circulation.^33^

Renal dysfunction as a result of the unphysiological Fontan circulation could also explain the observed association between baseline NT-proBNP z-score and eGFR_cr_ deterioration rate over time. N-terminal pro-brain natriuretic peptide is a widely used cardiac biomarker in the diagnosis and management of adults with heart failure and structurally normal, biventricular hearts, whereas in patients with a univentricular circulation, its interpretation is incipient. However, direct measurements of Fontan hemodynamics (eg, invasively measured CVP and CO) are often not part of the routine follow-up of these patients. Therefore, surrogate markers for circulatory performance of the Fontan circulation are needed. Recently, Ghelani et al reported a correlation between NT-proBNP and CMR-derived ventricular dilation and dysfunction.^34^ Both ventricular dilation and dysfunction are strong predictors of adverse events in Fontan circulations.^35^ Additionally, our group previously reported that NT-proBNP z-score was strongly associated with the extent of venous congestion, a key determinant of failure of the Fontan circulation.^11^ Furthermore, although NT-proBNP is cleared from the circulation by the kidneys, the association found between baseline NT-proBNP z-score and the rate of eGFR_cr_ deterioration was independent of serum cystatin C. Therefore, these findings suggest a potential role for NT-proBNP z-scores as a surrogate marker for the current status of the Fontan circulation and an early predictor for the imminent deterioration rate of renal function in patients with a Fontan circulation.

### Strengths and limitations

Our study has a few strengths and limitations. First, the longitudinal nature of this study makes it unique and allows us to more accurately investigate the influence of the Fontan circulation on renal function. Additionally, renal function was determined in the context of regular follow-up protocol, which decreases the chance of bias. Of note, the studied cohort has a relatively low incidence of patients with diagnosed hypoplastic left heart syndrome, which may limit the generalizability of the findings. Longitudinal measurements of GFR, either measured directly or calculated using serial measurements of serum cystatin levels, would have circumvented the discussed limitations of eGFR_cr_ and might have provided an even more accurate reflection of the deterioration of renal function over time. Also, a direct comparison of the course of eGFR_cr_ over time between Fontan patients and healthy individuals is statistically not possible due to the cross-sectional nature of the available reference data. The advantage of the cohort used, however, is the fact that it is a Dutch cohort of consecutive patients, which decreases the potential bias of ethnicity in the calculation of eGFR_cr_ by the CKD-EPI equation. Finally, there were only 8 deaths among patients with Fontan circulation, which limits the ability to examine factors associated with outcomes.

## Conclusions

In patients with Fontan circulation, the decline in renal function is more rapid than in healthy individuals. Using cystatin C instead of creatinine to estimate eGFR results in a higher estimate of the prevalence of renal dysfunction. A higher NT-proBNP z-score at baseline predicts a more rapid deterioration rate of eGFR_cr_. Lastly, a more rapid deterioration of eGFR_cr_ is associated with increased risk of mortality. These findings highlight the importance of ongoing surveillance for renal dysfunction in the Fontan population.

Perspectives **COMPETENCY IN MEDICAL KNOWLEDGE:** Multiorgan complications after the Fontan palliation are common. Renal dysfunction in the setting of multiorgan complications is often under-recognized. Although patients palliated with Fontan circulation may start their adulthood with an eGFR_cr_ that is comparable to that of healthy individuals, the deterioration of eGFR_cr_ is faster in Fontan patients.

**TRANSLATIONAL OUTLOOK:** Considering creatinine-based eGFR is prone to overestimate renal function in patients with Fontan circulation, the deterioration of renal function in this population could be even more severe than shown in this study. Additionally, renal dysfunction may be an early and progressive manifestation of a failing Fontan circulation, and this requires further study.

## Funding support and author disclosures

Dr Berger has UMCG contracts with Johnson & Johnson, MSD, Ferrer, and GSK for steering committee and advisory board activities of RMFB, outside the scope of this manuscript. All other authors have reported that they have no relationships relevant to the contents of this paper to disclose.
